# *Blastomycosis* Endocarditis: Case Report and Literature Review

**DOI:** 10.1093/ofid/ofad572

**Published:** 2023-11-15

**Authors:** Franco Murillo Chavez, Evgenii Filippov, Francesca Licandro, Vishal Sethi, Brandon Eilertson

**Affiliations:** Sinai Hospital of Baltimore, Baltimore, Maryland, USA; Facultad de Medicina Humana, Universidad Científica del Sur, Lima, Perú; Sinai Hospital of Baltimore, Baltimore, Maryland, USA; Sinai Hospital of Baltimore, Baltimore, Maryland, USA; Sinai Hospital of Baltimore, Baltimore, Maryland, USA; Kaiser Permanente Mid-Atlantic Permanente Medical Group, Maryland, USA

**Keywords:** blastomycosis, blastomycosis endocarditis, fungal endocarditis

## Abstract

We report successful treatment of a case of disseminated blastomycosis originating in the right lung, with involvement of the right pleural space, multiple ribs and vertebral bodies, and the pericardium and mitral valve endocarditis. The 22-year-old patient presented with a 13-month history of right lower lobe pneumonia associated with fevers, night sweats, rib pain, and 27-kg weight loss. Pathology examination revealed *Blastomyces* from multiple biopsies of inflammatory masses in the right thorax. After a 4-week induction with liposomal amphotericin followed by oral itraconazole, the patient had complete resolution of the clinical and laboratory findings of blastomycosis.

## Graphical Abstract

**Graphical Abstract:**
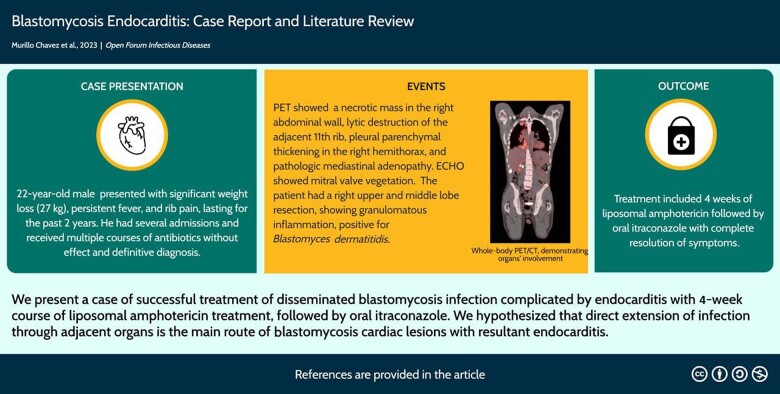

Blastomycosis is a fungal infection caused by the dimorphic fungi *Blastomyces* spp. Its main route of transmission is through direct inhalation of *Blastomyces* conidia [1]. After inhalation, it is converted into its yeast form within the host’s respiratory tissue, establishing infection. Moreover, conidia are phagocytized by lung macrophages, where they convert to the yeast form and can disseminate to any organ in the body [2]. The overall mortality of blastomycosis infection is between 4% and 22% [3]. Patients who progress to acute respiratory distress syndrome (ARDS) have mortality rates >50% [2]. Disseminated disease is seen in up to 40% of cases [4]. With a range of clinical presentations mimicking more common infections, delayed diagnosis of *Blastomyces* infection is common, increasing morbidity and mortality [5]. Blastomycosis with cardiac involvement is extremely rare, and there have been only 4 other case reports of *Blastomyces* causing infective endocarditis. In this report, we present a case of blastomycosis causing native valve fungal endocarditis that was successfully treated using antifungal therapy alone, with no surgical intervention.

## CASE PRESENTATION

We present the case of a 22-year-old male Maryland resident with a 27-kg weight loss, persistent fever, and rib pain. This presentation led to the identification of chronic pulmonary infiltrates, pleural effusion, subcarinal mass, lytic destruction of the 11th right rib, and a right flank abdominal wall abscess.

On initial presentation, he had a 2-week history of right-sided pleuritic chest pain, initially felt to be musculoskeletal in origin. On follow-up, he had a dry cough, and he completed 7 days of amoxicillin-clavulanate. At the same time, he developed chills each evening. He presented again 6 weeks later with worsening, persistent right chest pain that was pleuritic in nature. Computed tomography (CT) of the chest showed right lower lobe consolidation and small right pleural effusion plus an enlarged subcarinal lymph node. He was given azithromycin for 4 days and amoxicillin for 10 days. He felt slight improvement while on treatment. He returned 3 weeks later for worsening pain and cough. Repeat CT showed increased consolidation of the right middle and lower lobes of the lung and a large pleural effusion. He was admitted to the hospital and treated with levofloxacin and metronidazole. On further history, he had recent travel to Florida, and he worked at a home improvement retail store, where he moved mulch and dirt daily. He denied bird or animal exposure. He had 2 thoracenteses showing an exudative effusion with a lymphocytic predominance, negative bacterial and fungal cultures, plus negative acid-fast bacilli stains and culture. Flow cytometry and cytology showed no evidence of malignancy. He completed a 3-week course of oral levofloxacin and metronidazole for presumptive community-acquired pneumonia complicated with a parapneumonic effusion. He had resolution of symptoms on follow-up, and pleural effusion was insufficient for repeat thoracentesis 1 month later.

**Figure 1.**
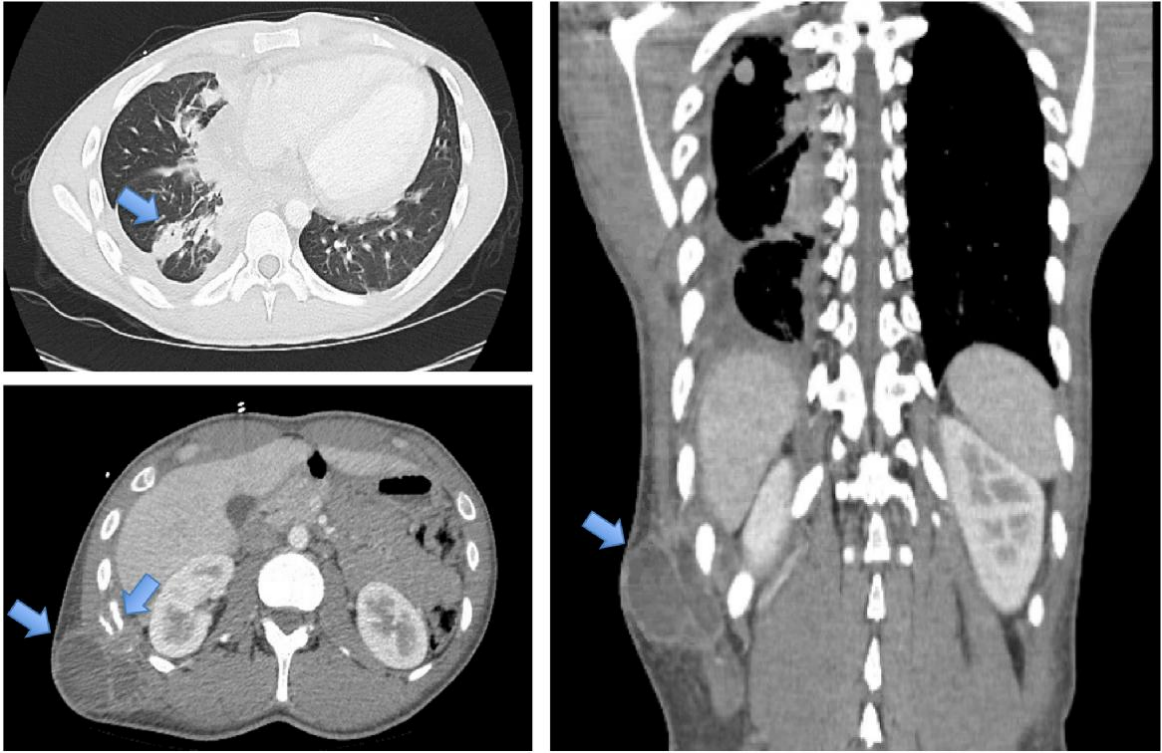
Chest and abdomen CT scan, demonstrating pulmonary, osseous, and cutaneous involvement. Abbreviation: CT, computed tomography.

**Figure 2.**
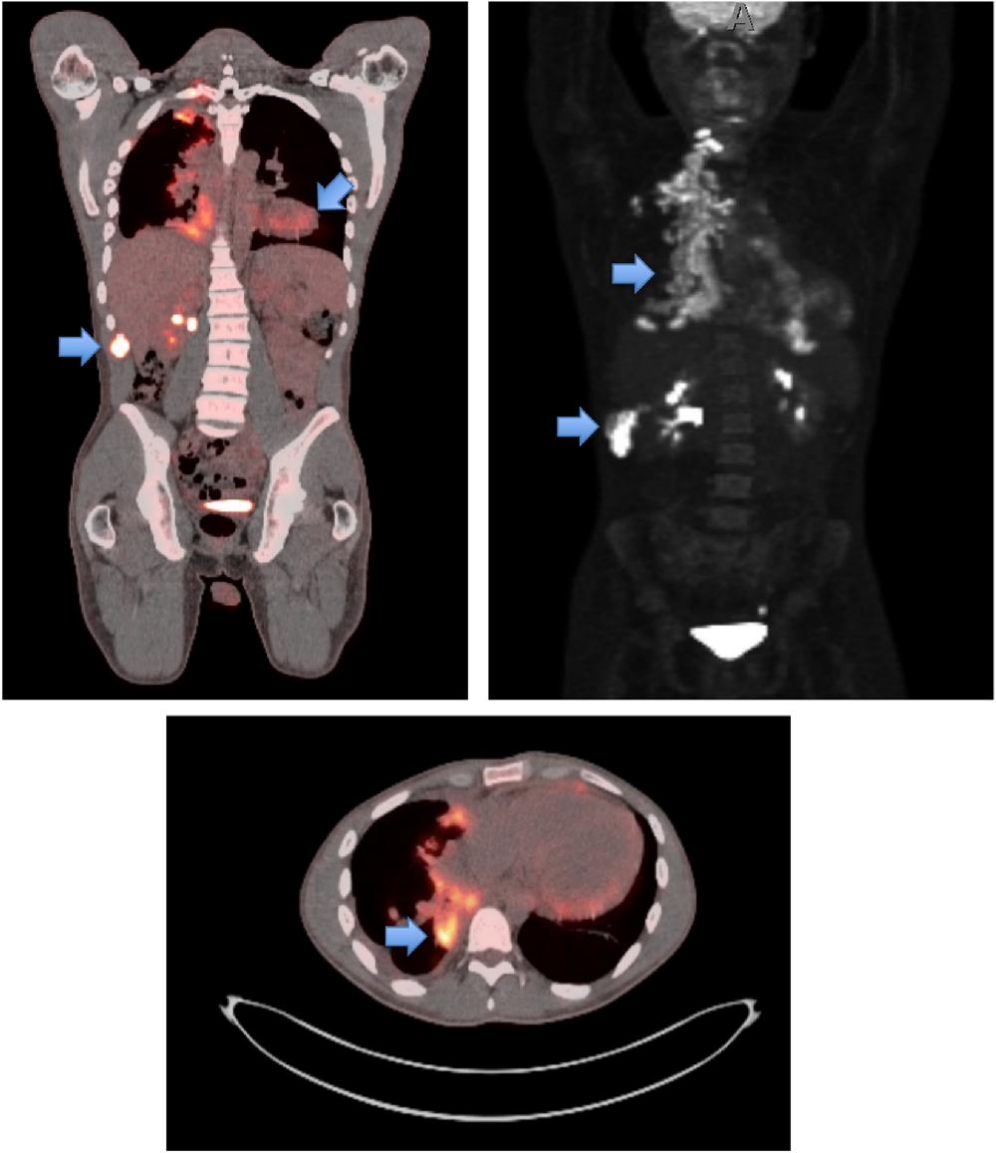
Whole-body PET/CT, demonstrating pulmonary, osseous, and cardiac involvement. Abbreviation: PET/CT, positron emission tomography/computed tomography.

**Figure 3.**
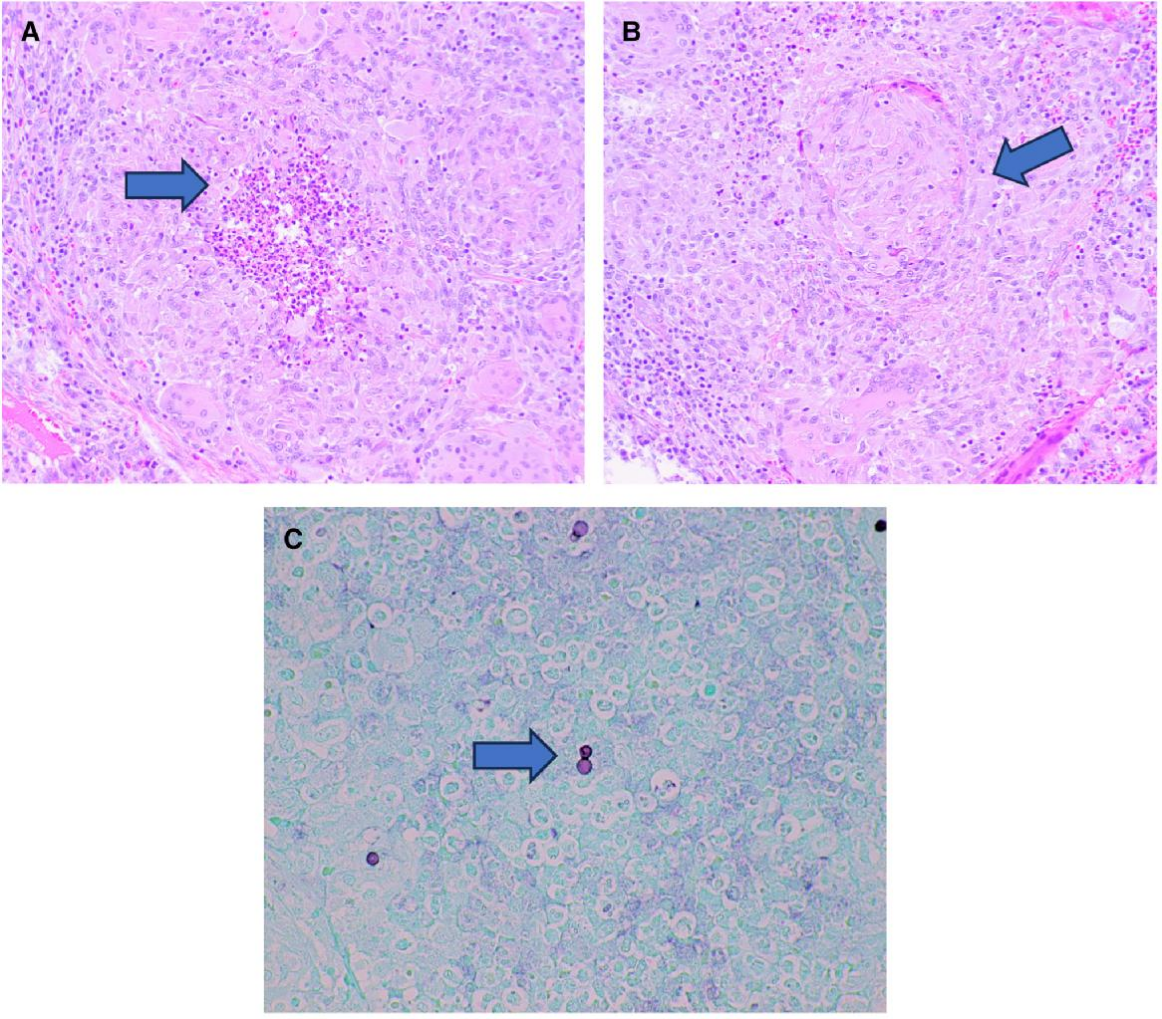
*A*, H&E-stained section of lung parenchyma with granulomatous inflammation and suppurative necrosis (200× magnification). *B*, H&E-stained section of lung parenchyma with granulomatous inflammation (200× magnification). *C*, GMS stain showing broad-based budding yeast forms (400× magnification). Abbreviations: GMS, Grocott-Gomori methenamine silver; H&E, hematoxylin and eosin.

**Figure 4.**
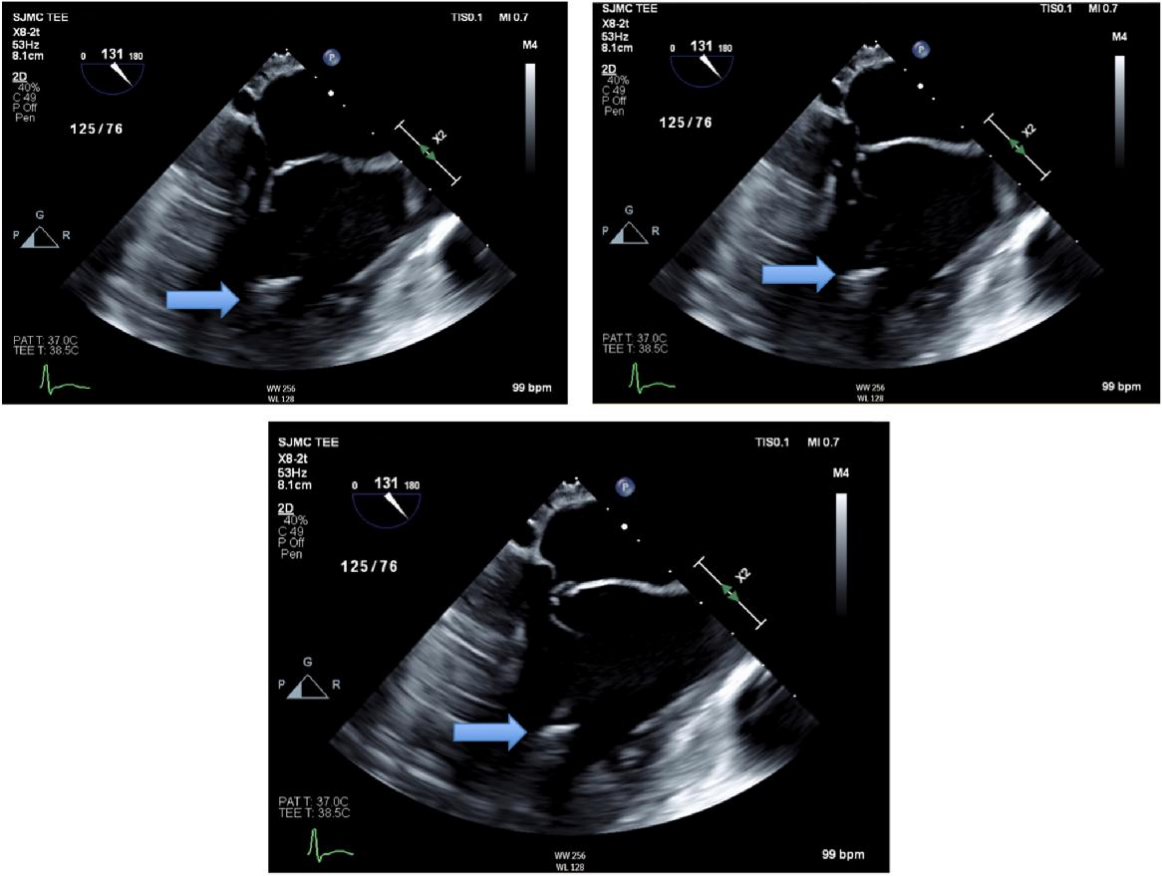
Transesophageal echocardiogram showing 1.5-cm filamentous mobile mass on the ventricular side of the mitral valve leaflet, suggestive of vegetation.

He presented to the hospital 12 months later with weight loss, fevers, and shortness of breath. CT and PET CT showed an abscess within the right flank abdominal wall with lysis of the adjacent right 11th rib and multifocal mass-like pleural parenchymal thickening within the right hemithorax and adjacent right hilum. Pathologic mediastinal adenopathy was also noted, as well as mild pericardial thickening with trace pericardial effusion and a small partially loculated right pleural effusion (Figure 1 and Figure 2). Thoracentesis revealed a lymphocytic predominant exudative effusion, with small lymphocytes and reactive mesothelial cells seen on cytology. He had a right upper and middle lobe wedge resection and decortication of pleura, and a biopsy showed fungal elements. Unfortunately, the skin abscess was drained spontaneously, and cultures were not obtained. There was concern for malignancy until pleural biopsies demonstrated non-necrotizing and necrotizing granulomatous inflammation with Grocott's methenamine silver stain and periodic acid-Schiff stains showing occasional broad-based budding yeast forms within the granulomas (Figure 3). Results of pathology were delayed, and the patient was discharged on oral antibiotics. Subsequently, a fungal culture from the biopsy identified *Blastomyces dermatitidis* (Mayo Medical Laboratories, Rochester, MN, USA), and the patient was started on liposomal amphotericin 5 mg/kg daily 3 weeks after surgery. Urine *Blastomyces galactomannan* was positive at 3.48 ng/mL (Miravista Diagnostics). The patient was subsequently readmitted for infusion reaction to amphotericin and fevers. In the setting of persistent fevers, a transthoracic echocardiogram was also obtained, which was suspicious for mitral valve vegetation. Subsequent transesophageal echocardiogram demonstrated a 1.5-cm filamentous mobile mass on the ventricular side of the mitral valve leaflet, suggestive of vegetation. TEE showed an ejection fraction of 55%–60% and no mitral regurgitation (Figure 4). Cardiology and thoracic surgery were consulted, and given the lack of other indications for surgery, conservative management was continued. Fevers resolved, and he was discharged home to complete liposomal amphotericin 5 mg/kg daily for 4 weeks followed by oral itraconazole 200 mg orally 3 times daily for 3 days then twice daily for a 1-year course of treatment. Upon further investigation, he was noted to have a CD4 count of 89/16%, despite negative HIV antibody and undetectable HIV RNA PCR. Two months into therapy, repeat CD4 improved to 551/36%, and immunologic workup showed normal CH50 plus normal levels of immunoglobulin (Ig)A, IgG, IgE, and IgM. B, T, and natural killer cell levels were in the normal range. By 3 months into treatment, the patient reported complete resolution of dyspnea and cough, even with heavy exertion. The patient declined repeat echocardiogram at 3 months into the treatment course.

## DISCUSSION

Blastomycosis is a fungal infection that is usually encountered in North America. It is endemic to the Eastern half of the United States and Canada, with the highest incidence in the North-Central states. The first mention of cardiac lesions caused by *Blastomyces* dates to 1916, when Hurley described invasion of the pericardium, myocardium, and endocardium by *Blastomyces* [6]. In 1937, Baker postulated that these pathological findings might result from an extension from adjacent structures such as the lungs or bones [7]. Both these authors, along with Pond et al., described patients with full-thickness heart injuries who also had solitary and multiple nodules in the endocardium, presumed to be associated with *Blastomyces*. Notably, there was no mention of valvular disease or further reference to endocarditis in these cases [6–8]. Although the primary mechanism typically involves bloodstream fungemia originating from the lungs and skin, leading to secondary seeding of the mitral valve, we concur with Baker and hypothesize that contiguous spread could provide an alternative explanation. In our patient, the infection began in the right lung and first spread through the pleura into the mediastinum, which extended through the pericardium, causing effusion. We also believe that the fungal infection affected the full thickness of the heart wall, which extended to the endocardial surface of the mitral valve area. The patient's positron emission tomography scans (Figure 3) further support this hypothesis, aligning with the natural course of untreated blastomycosis, which can disseminate through virtually any tissue after surviving phagocytosis.

Dimorphic fungal endocarditis is considered extremely rare in comparison with other fungal etiologies such as *Candida* and *Aspergillus* spp. [9]. *Histoplasma* endocarditis has been described the most frequently, with 80 cases [10], followed by a few reported cases of *Coccidiodes* and single cases of *Paracoccidioides* and *Sporothrix* endocarditis [9, 11]. The valve most often affected is the aortic in *Histoplasma* endocarditis, vs the mitral valve with *Coccidioides* [9, 11].

In 2 prior case reports, blastomycosis endocarditis affected native tricuspid valves, unlike our case where the mitral valve was affected (Table 1).

**Table 1.** Demographics and General Characteristics of Reported Blastomycosis Endocarditis Cases

Patient	Age, y	Sex	Comorbidities/Immunosuppression	Organ Involvement	Valve	Treatment	Outcome	Ref
1	21	M	No	Lung, bone, skin, and heart	Native mitral	Amphotericin B + itraconazole	Alive	Our case
2	21	F	Yes (pregnant)	Lung, skin, heart	Native tricuspid	Amphotericin B + itraconazole	Alive	Waker et al. [10]
3	20	F	Yes (therapy for juvenile arthritis)	Lung, heart	Native tricuspid	Antigungal for disseminated blastomycosis	Alive	Richards et al. [12]
4	31	M	Yes (alcoholic liver cirrhosis, dilated cardiomyopathy, chronic pancreatitis)	Lung, heart	No valve was affected	Amphotericin B	Death	Alhaji et al. [13]
5	33	M	No	Lung, heart	Nonvalvular endocarditis, a nodule on atrial surface	Penicillin (presumed bacterial endocarditis)	Death	Pond and Humphrey [14]

Due to its rarity, treatment of *Blastomyces* and other dimorphic fungi-induced endocarditis relies on case report–level data. In cases of *Coccidioides* and *Histoplasma* endocarditis, the best outcomes have been achieved when surgery and antifungal therapy were combined [15]. The mortality rate among 7 cases of coccidioidomycosis and 52 cases of histoplasmosis was 100% and 50% if treated only with antifungals vs valve replacement with concomitant medical therapy [9, 16]. However, in the cases of blastomycosis endocarditis, antifungal therapy alone achieved a success rate in the totality of the reported cases (Table 1). In the present case, the patient was hemodynamically stable and lacked typical indications for valve replacement, so he was treated medically with liposomal amphotericin B induction followed by itraconazole, with a favorable outcome.

Amid increased reports of dimorphic fungal endocarditis, case incidence of dimorphic fungal infection has increased outside historic endemic ranges [6]. Clinicians should consider endemic mycoses in their differential diagnosis even outside of areas of classical geographic risk [7]. This is particularly important because diagnostic delays are common. For instance, a study by Lemos et al. found that out of 123 patients ultimately diagnosed with blastomycosis, only 18% were initially suspected of having it [8]. Aysun et al. noted that diagnostic delay for pulmonary blastomycosis was common, which they attributed to an overall “lack of consideration” of *Blastomyces* in favor of other diagnoses [3]. In their study, they mentioned that patients were treated with antibacterial agents and corticosteroids before further testing. Furthermore, they suggested that novel approaches need to be investigated to aid with earlier detection [3]. Additionaly, Suneja pointed out that the diagnosis of blastomycosis is generally delayed for >1 month in 40% of cases, with a moderate to major impact on clinical care in about 66% of cases [6, 15]. Interestingly, our patient was considered to have pneumonia, malignancy, and tuberculosis on his preliminary differential diagnosis; these are considered the most frequent alternative diagnoses [8]. Our case also experienced a delay in diagnosis, which likely contributed to the development of his rare presentation of *Blastomyces* endocarditis.

## CONCLUSIONS

We present a case of successful treatment of disseminated blastomycosis infection complicated by endocarditis. Blastomycosis with cardiac involvement is rare, and endocarditis is extremely rare. While bloodstream fungemia could be a primary mechanism, we hypothesized that direct extension of infection through adjacent organs is the main route of blastomycosis cardiac lesions with resultant endocarditis. The patient received a 4-week course of liposomal amphotericin treatment, followed by oral itraconazole, resulting in complete resolution of symptoms. Finally, the diagnosis of blastomycosis should be considered even in non–traditionally endemic areas as it is a frequently missed diagnosis and could have major implications on public health.
